# Placenta increta in an unscarred and bicornute uterus

**DOI:** 10.12669/pjms.39.1.6164

**Published:** 2023

**Authors:** Namia Nazir, Daniya Khan, Farah Deeba Nasrullah, Riffat Jaleel

**Affiliations:** 1 Dr. Namia Nazir Postgraduate Trainee, Gynae Unit III, Dow University of Health Sciences (CHK), Karachi, Pakistan; 2 Dr. Daniya Khan Postgraduate trainee, Gynae Unit III, Dow University of Health Sciences (CHK), Karachi, Pakistan; 3 Dr. Farah Deeba Nasrullah Associate Professor, Gynae Unit III, Dow University of Health Sciences (CHK), Karachi, Pakistan; 4 Prof. Riffat Jaleel Head of Department (OBGYN), Gynae Unit III, Dow University of Health Sciences (CHK), Karachi, Pakistan

**Keywords:** Placenta Increta, Unscarred uterus, Bicornute uterus

## Abstract

Morbidly adherent placenta is a spectrum of obstetric complication which is life threatening to both mother and fetus. Congenital uterine malformation is a rare cause of such a condition. Here we present a case report of placenta increta in bicornute, unscarred uterus. An 18 year old para1+1 presented in emergency with history of vaginal delivery of still birth baby followed by vaginal bleeding with retained placenta. Her Examination under anaesthesia and failed attempt of manual removal of the placenta performed in emergency followed by Doppler ultrasound showed a bicornuate uterus with possibility of placenta increta, later this diagnosis was confirmed on magnetic resonance imaging (MRI). Patient managed with injection methotrexate along with folinic acid followed by removal of placenta under general anesthesia, hence we preserved her fertility. The aim of this report is to emphasize the importance of this rare but a possible association of nonscar and malformed uterus with spectrum of abnormal placentation. Obstetrician should run a full set of investigations in such cases to prevent maternal and fetal mortality and morbidity.

## INTRODUCTION

Morbidly adherent placenta is a condition of abnormal placentation in which trophoblastic cell invades into myometrium rendering the placenta unable to detach from the uterine wall causing massive antepartum or postpartum hemorrhage and significant maternal and fetal mortality. Depending on the degree of invasion, this condition can be presented in any of these three conditions.^1^,^2^

### Placenta Accreta:

When the placental chorionic villi is attached directly with the myometrium.

### Placenta Increta:

When placental chorionic villi invades into the myometrium.

### Placenta Percreta:

When placental chorionic villi invades through the uterine wall, in some condition may permeate to close by organs like bladder or bowel.

The risk factors of morbidly adherent placenta include previous caesarean sections, previous myomectomy, previous uterine evacuation and uterine anaomolies.^3^,^4^ We report a case of morbidly adherent placenta in the women having unscarred, bicornute uterus who presented with vaginal bleeding and retained placenta after the vaginal delivery of preterm still birth baby. She was successfully managed conservatively with preservation of fertility. This should alarm the obstetrician that all those patient who has a history of uterine evacuation or have any mullerian malformation should undergo a screening test via gray scale Doppler ultrasound and if needed MRI should be done to avoid such maternal and fetal morbidities and possible mortalities as well.

## CASE PRESENTATION

An 18 year old patient, para 1+1, was brought to emergency of our hospital with the history of delivery of still birth baby three hours ago at home by traditional birth attendant (Dai) at 30 weeks of gestation followed by vaginal bleeding and retained placenta. Her previous pregnancy was ended spontaneously in first trimester miscarriage followed by instrumentation by traditional birth attendant that was uneventful. In index pregnancy her antenatal period was not followed except for a dating scan that was done at nine weeks of gestation. She went into preterm labor at 30 week of gestation and was attended by traditional birth attendant at home, which ended up in vaginal birth of still born baby boy and retained placenta. After one hour she was referred to tertiary care hospital.

**Fig.1:**
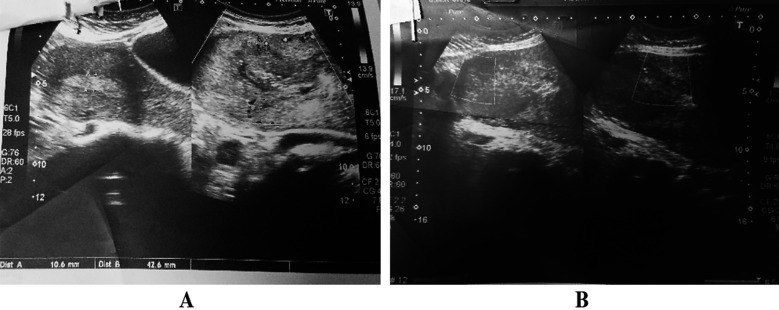
Doppler ultrasound showing bicornuate uterus (a) with placental invasion within myometrium(b).

**Fig.2:**
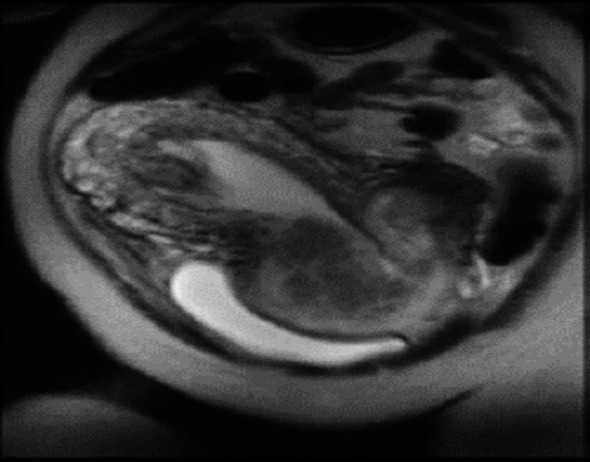
MRI showing bicornuate uterus with placenta increta

In Emergency patient was received in hemodynamically stable state with GCS of 15/15, blood pressure of 110/70mmHg, Pulse rate of 100 beats/mins. After initial stabilization she was shifted to operation theater for manual removal of placenta (MRP). We were unable to remove the placenta as cleavage plane was not found, she remained hemodynamically stable throughout, therefore procedure was abandoned. Doppler ultrasound (Figure 1) showed bicornuate uterus with placenta in situ measuring 7×8cm attached and invading the lower uterine segment, suggestive of placenta increta. Her MRI was performed for confirmation of diagnosis that showed the same findings (Fig.2). As patient was hemodynamically stable and had no alive baby before and patient and her relatives wanted to preserve her fertility, we decided to manage her conservatively. After base line Full blood count, Liver function test and Renal function test, were done Injection methotrexate 50mg intramuscularly was given followed by Injection Folinic acid 15mg to counteract methotrexate toxicity on alternate days. Three doses were given. She was kept on broad spectrum I/V antibiotics and monitored for signs of infection by monitoring pulse, temperature and uterine tenderness and serial CBC and CRP levels. Patient remained afebrile during her stay at hospital. Her blood cultures and High vaginal swabs did not show any infection hence conservative management was continued. She was also monitored for signs of methotrexate toxicity by history and performing CBC, RFTs and LFTs. After three doses of methotrexate she started having mild bleeding per vaginum. Doppler ultrasound was repeated that showed reduction in placental size to 5×3 cm so hysteroscopy and manual removal of placenta was planned and performed successfully. A week later followed up scan was done that showed no placental remnant, hence successfully managed to preserve her fertility.

## DISCUSSION

The incidence of morbidly adherent placenta is reported as 2-90/10000 live births and according to a study the incidence rate was 0.017%.^5^ Among the type of abnormally invasive placenta, placenta accrete stands at the top, occurs in around 75% of all case, however placenta increta accounts for 18% and placenta percreta around 7%.^6^,^7^ Perinatal mortality occurs in around 8% and hysterectomy needs to be performed in around 13 % of cases.^1^

As already discussed there are many risk factor for abnormally adherent placenta among which previous caesarean section is the most common cause, other causes are previous myomectomy, multiparity, previous manually removed placenta, placenta previa , septic endometritis , any uterine malformation.^8^

Antenatal diagnosis and anticipation of risk factor is a crucial step in managing such obstetrics complications as undiagnosed case leads to complications like retained placenta, exsanguinating postpartum hemorrhage, sepsis, maternal and fetal mortality.^9^

Screening with Doppler ultrasonography and magnetic resonance imaging is very helpful in the early diagnosis of morbidly adherent placenta but the final diagnosis is based on histological examination.^10^

The role of methotrexate in treating morbidly adherent placenta remains controversial because of its uncertain function and risk of adverse side effects like, pancytopenia, nephrotoxicity, hepatotoxicity, immunosuppression and gastrointestinal upsets. It is hypothesized that methotrexate acts by inducing placental necrosis causing rapid involution of placenta though there is a lack of consensus for the optimum dose, frequency and timing of administration of methotrexate.

However, many small studies have shown promising results of methotrexate in the management of morbidly adherent placenta. This method was chosen for the patient due to strong desire of preservation of fertility by patient and her family. Though the treatment was successful without any complication, there is a need to have studies with adequate sample size before referring this management as preferred choice for morbidly adherent of placenta.

In this case report patient has presented with rare risk factors of this condition like having of history of previous single uterine evacuation and undiagnosed uterine malformation. Case handling by traditional birth attendant and lack of antenatal care seems to be the primary reason for complications. As most of such patients need hysterectomy but fortunately in this case conservative management succeeded by methotrexate chemotherapy along with injection folinic acid and her fertility was preserved. Doppler ultrasonography can be considered for screening abnormal placentation in pregnant women having congenital uterine malformation or past history of surgical intervention for optimal maternal and fetal outcome.

## CONCLUSION

Abnormally invasive placenta is a life threatening condition to both mother and fetus. Increasing incidence makes screening of all the potential high risk pregnancies necessary for their appropriate management. Awareness regarding the common and rare risk factor is very crucial in early detection and prevention of morbidity and mortality associated with this condition.

### Authors’ Contribution:

**NN, DK:** Selection of case, Designed, writing, Editing of manuscript.

**FDN, RJ:** Supervision of Management Plan, Final review and approval of case report.
